# Unveiling CO_2_ heterogeneous freezing plumes during champagne cork popping

**DOI:** 10.1038/s41598-017-10702-6

**Published:** 2017-09-14

**Authors:** Gérard Liger-Belair, Daniel Cordier, Jacques Honvault, Clara Cilindre

**Affiliations:** 1 Equipe Effervescence, Champagne et Applications (GSMA - UMR CNRS 7331), Université de Reims Champagne-Ardenne, UFR Sciences Exactes et Naturelles, BP 1039, 51687 Reims Cedex 2, France; 2 Engineering Art, 17 rue Bel Air, 60110 Amblainville, France

## Abstract

Cork popping from clear transparent bottles of champagne stored at different temperatures (namely, 6, 12, and 20 °C) was filmed through high-speed video imaging in the visible light spectrum. During the cork popping process, a plume mainly composed of gaseous CO_2_ with traces of water vapour freely expands out of the bottleneck through ambient air. Most interestingly, for the bottles stored at 20 °C, the characteristic grey-white cloud of fog classically observed above the bottlenecks of champagne stored at lower temperatures simply disappeared. It is replaced by a more evanescent plume, surprisingly blue, starting from the bottleneck. We suggest that heterogeneous freezing of CO_2_ occurs on ice water clusters homogeneously nucleated in the bottlenecks, depending on the saturation ratio experienced by gas-phase CO_2_ after adiabatic expansion (indeed highly bottle temperature dependent). Moreover, and as observed for the bottles stored at 20 °C, we show that the freezing of only a small portion of all the available CO_2_ is able to pump the energy released through adiabatic expansion, thus completely inhibiting the condensation of water vapour found in air packages adjacent to the gas volume gushing out of the bottleneck.

## Introduction

Uncorking a bottle is indeed the first action preceding champagne tasting, and even if it is far safer and advised to uncork a bottle of champagne with a subdued sigh, anyone of us has certainly already experienced popping the cork with a bang^1, 2^. From a strictly physicochemical point of view, Champagne wines are multicomponent hydroalcoholic systems, with a density close to unity, a surface tension close to $50\,{{\rm{m}}{\rm{N}}{\rm{m}}}^{-1}$ (indeed highly ethanol-dependent), and a viscosity about 50% higher than that of pure water (also mainly due to the presence of 12–13% v/v ethanol)^3^. Moreover, Champagne and sparkling wines elaborated through the same traditional method hold a concentration of dissolved carbon dioxide (CO_2_) formed together with ethanol during a second fermentation process (called *prise de mousse*) promoted by adding yeasts and a certain amount of sugar in the sealed bottles. It is an application of Henry’s law which states that the concentration of dissolved CO_2_ in the liquid phase is proportional to the partial pressure of gas-phase CO_2_ above the solution, in the bottleneck. Nevertheless, the solubility of CO_2_ in the liquid phase being strongly temperature-dependent, the partial pressure of gas-phase CO_2_ in the bottleneck is therefore also strongly temperature-dependent. During the cork popping process, a plume mainly composed of gaseous CO_2_ with traces of water vapour freely expands out of the bottleneck through ambient air. Recently, the cloud of gaseous CO_2_ expanding out of the bottleneck while cork popping, was made visible through high-speed infrared imaging^4^. Differences according to the bottle’s temperature were clearly observed concerning both the volume of gas-phase CO_2_ gushing out of the bottleneck, and its overall dynamics. Results were discussed by considering that gases under pressure in the bottleneck experience adiabatic expansion while cork popping. Observations of the cork popping process were already conducted in visible light with green bottles of sparkling wine stored at a single temperature on the order of 10 °C, and therefore at a single corresponding pressure in the order of only 5 bar^5^. Condensation of water vapour above the bottleneck was clearly observed during the cork popping process, thus revealing the rapid cooling of ambient air packages adjacent to the flow of gas gushing out of the bottleneck. Nevertheless, the opacity of classical dark green opaque glass bottles used in the previous set of experiments forbade the visualization of condensation processes in the bottlenecks^5, 6^.

Here high-speed video imaging was used to visualize cork popping, and especially the condensation processes following the adiabatic expansion of the gas mixture found in the bottlenecks of transparent champagne bottles stored at different temperatures. The bottle temperature was found to be a key parameter concerning the condensation processes that can occur above, and inside the bottlenecks. After adiabatic expansion of the gas mixture, and on the basis of the respective saturation ratios and corresponding homogeneous nucleation rates of the gas species initially present in the bottleneck before cork popping (mainly composed of gas-phase CO_2_ with traces of water and ethanol vapours), a scenario was proposed that explains our visually appealing observations.

## Results

**Figure 1:**
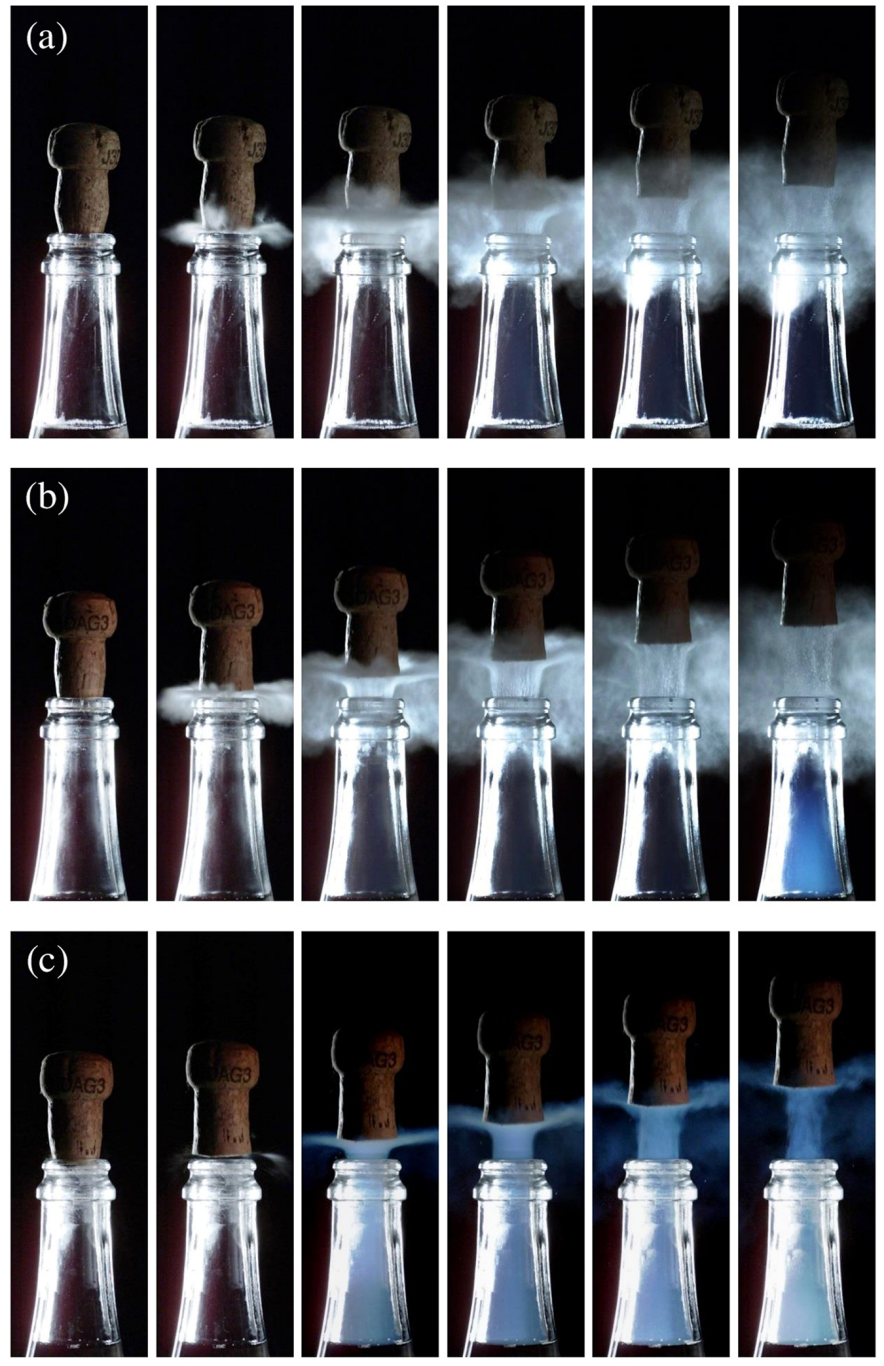
Time sequences showing the cork popping process from bottles stored at three different temperatures, namely 6 °C (**a**) 12 °C (**b**) and 20 °C (**c**). The time interval between each frame is 400 µs.

**Figure 2:**
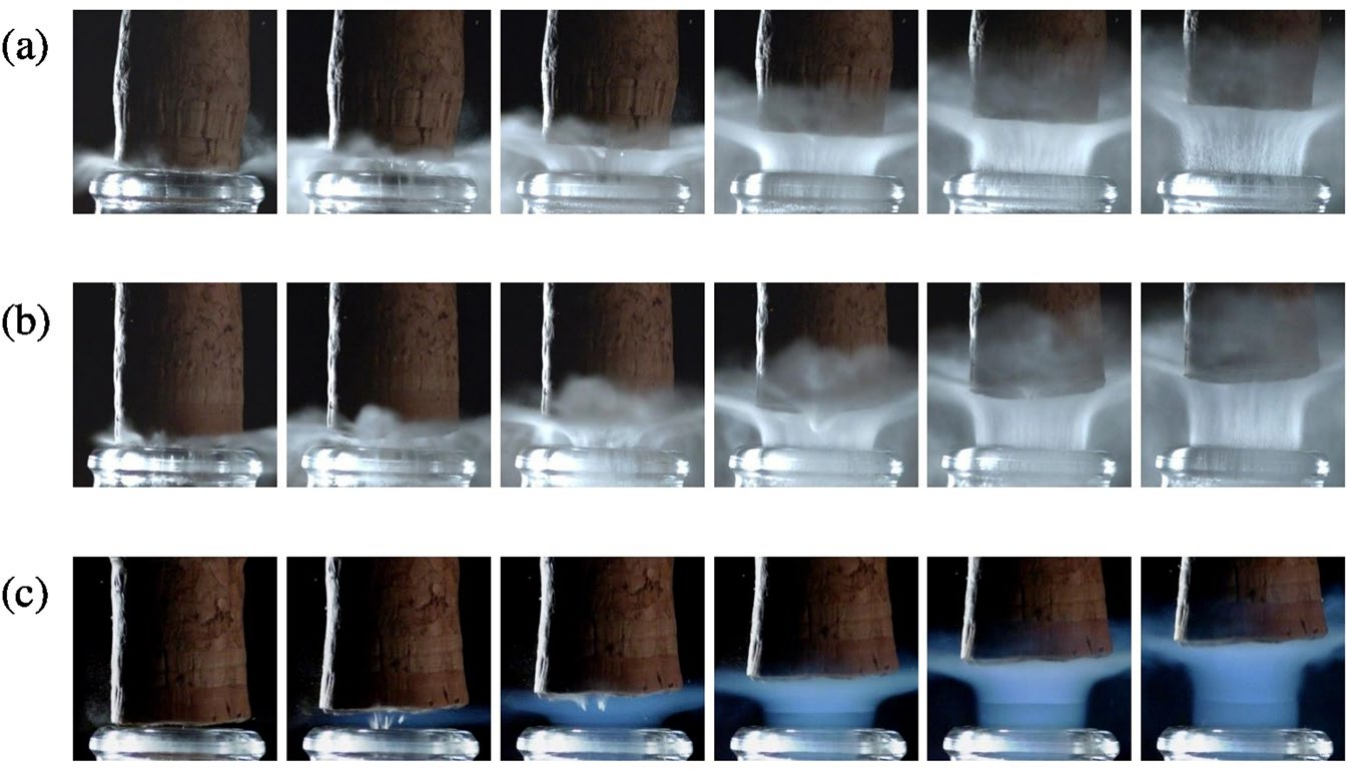
Close-up time sequences showing details of the cork popping process just above the bottlenecks of bottles stored at three different temperatures, namely 6 °C (**a**), 12 °C (**b**), and 20 °C (**c**). The time interval between each frame is 167 µs.

**Figure 3:**
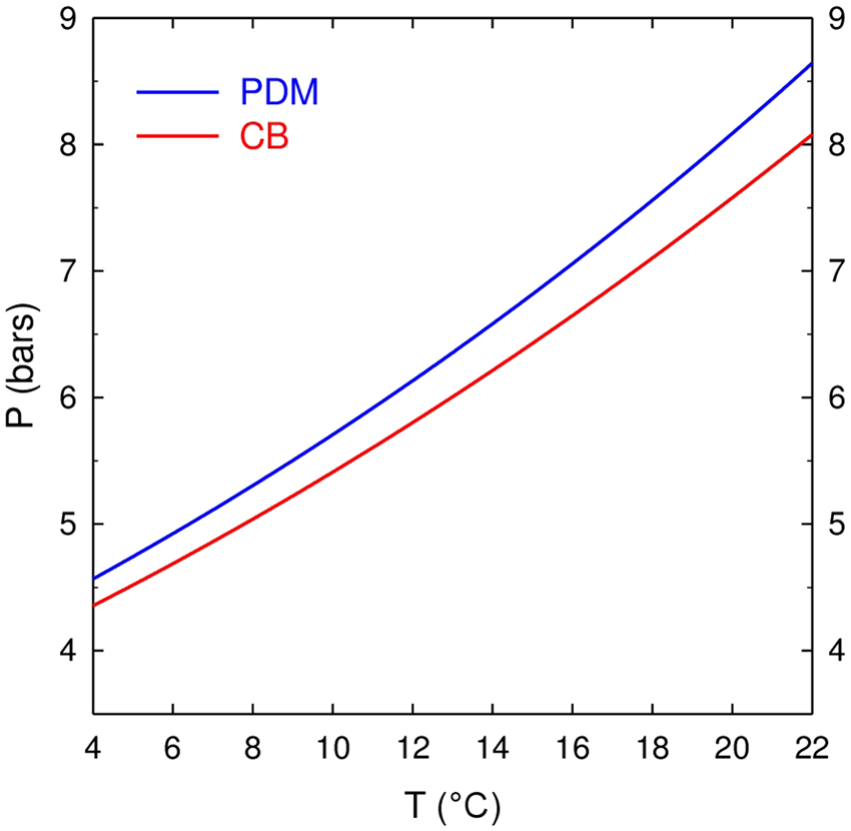
Partial pressure of gas-phase CO_2_ inside the sealed bottle as a function of champagne temperature, as determined through equations (13) and (15). The blue line corresponds to the gas-phase CO_2_ pressure reached after the *prise de mousse* in the bottles sealed with a cap, namely ${P}_{{\rm{PDM}}}^{{{\rm{CO}}}_{2}}$, whereas the red line corresponds to the equilibrium gas-phase CO_2_ pressure reached after the disgorging process in the corked bottles ready for cork popping, namely ${P}_{{\rm{CB}}}^{{{\rm{CO}}}_{2}}$.

Time-sequences displayed in Figs 1 and 2 illustrate champagne cork popping as seen through high-speed video imaging, for bottles stored at 6 °C, 12 °C, and 20 °C, respectively. As already observed previously, the cork popping process is characterized by the formation of a cloud of fog above the bottlenecks^2, 5, 6^. Contrary to popular belief, this cloud of fog is not formed by gas-phase CO_2_ gushing out of the bottleneck, indeed invisible in the visible light spectrum, and made visible only through infrared imaging^4^. The commonly accepted idea is that the gas mixture trapped in the headspace under the cork experiences a drop of pressure while cork popping, from the pressure inside the bottle before uncorking - strongly temperature dependent, as seen in Fig. 3 - to the ambient pressure close to 1 bar. Details about the temperature dependence of the pressure found inside the corked bottles can be found in the Methods section. Assuming adiabatic expansion, a corresponding huge drop of temperature experienced by the gas mixture gushing out of the bottleneck therefore also inevitably cools adjacent air packages (before thermal equilibrium at room temperature is re-established again), thus causing the condensation of water vapour found in ambient air. High-speed time-sequences showing the cork popping process at 6 and 12 °C, as displayed in Fig. 1a,b, clearly show a grey-white cloud of fog right above the bottlenecks. The grey-white colour is characteristic of Mie scattering (i.e., the scattering of light by particles with typical sizes larger than the wavelength), well known to be at the origin of cloud colour in the sky^7^. It is worth noting that these very characteristic grey-white plumes occurred only outside the bottleneck. Actually, a previous set of experimental observations in dry atmosphere clearly showed the absence of such a white plume above the bottleneck of freshly uncorked bottles of champagne^5^, thus supporting the idea that the condensation of the water vapour found in ambient air is indeed responsible for such a grey-white fog right above the bottlenecks.

Most interestingly, bottles stored at 20 °C showed a very different behaviour. The characteristic grey-white cloud of fog observed above the bottlenecks of champagne stored at 6, and 12 °C (rather isotropically oriented), simply disappears, and is replaced by an even more evanescent plume, vertically oriented, starting from inside the bottleneck, and surprisingly blue (see Figs 1c and 2c). Blue haze is indeed characteristic of processes controlled by Rayleigh scattering (i.e., the scattering of light by particles with typical sizes much smaller than the wavelength). Contrary to the grey-white fog forming exclusively right above the bottlenecks, the blue haze accompanying the cork popping process starts inside the bottlenecks, and therefore in a region almost exclusively composed of gaseous CO_2_. Otherwise, it is interesting to mention that blue haze can also be observed in the bottlenecks of champagne stored at 12 °C (about 2 ms after the grey-white fog has formed above the bottlenecks – see Fig. 1b), whereas for bottles stored at 20 °C, blue haze appears more rapidly (about 0.8 ms after cork popping – see Fig. 1c). Moreover, it is noteworthy to mention that blue haze has never been observed inside the bottleneck during the cork popping process of bottles stored at 6 °C.

The same scenario was strictly observed for each bottle belonging to a given batch of bottles stored at a given temperature. We are logically tempted to wonder why such striking differences were observed regarding the dependence with the initial bottle temperature of both the colour, and the overall dynamics of condensation processes that accompany the cork popping process.

## Discussion

### A discussion based on adiabatic expansion

Adiabatic expansion and its drop of temperature while cork popping, is undoubtedly the mechanism behind the condensation processes observed above and within the bottlenecks. The drop of temperature experienced by the gas mixture gushing out of the bottleneck while cork popping classically obeys the following equation:

1 $${T}_{f}=T\times {(\frac{{P}_{0}}{{P}_{CB}})}^{\frac{\gamma -1}{\gamma }}$$

with *T* and ${P}_{CB}$ being the temperature and pressure of gas phase before cork popping (in the sealed bottle), ${T}_{f}$ and ${P}_{0}$ being the final temperature and pressure of gas phase after adiabatic expansion, and *γ* being the ratio of specific heats of the gas phase experiencing adiabatic expansion (mainly composed of gaseous CO_2_ and being equal to 1.3)^8^.

**Figure 4:**
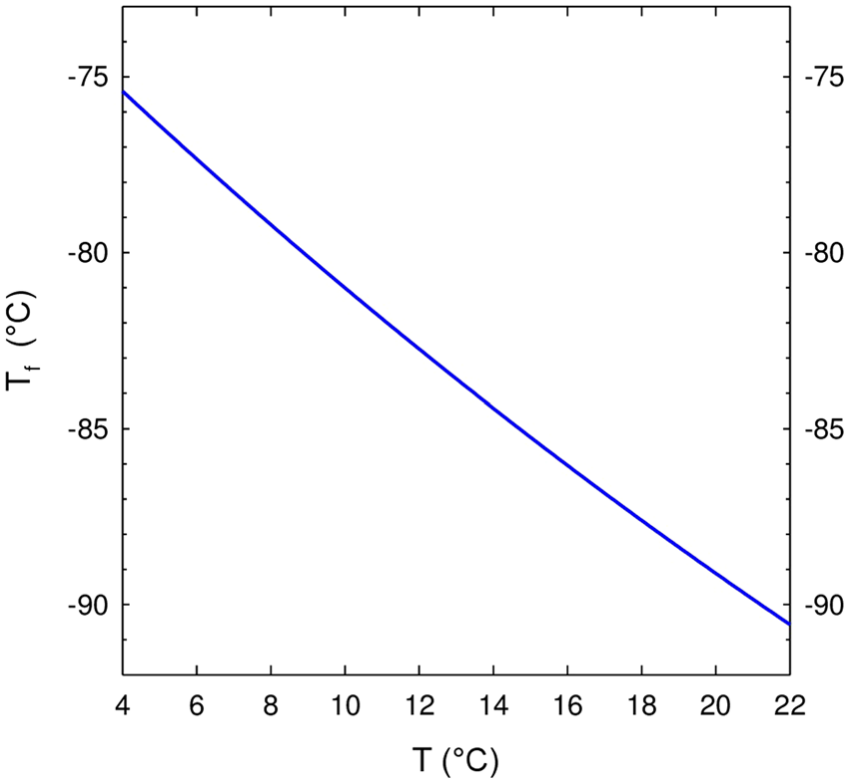
Final temperature ${T}_{f}$ reached by the gas mixture (mostly composed of gas-phase CO_2_) gushing out of the bottleneck after adiabatic expansion, as a function of the initial storage temperature *T* of champagne.

By combining equations (1) and (15) (which provides the initial pressure of gas-phase CO_2_ within the sealed bottle, before cork popping), and by replacing each and every parameter by its numerical value, the final temperature ${T}_{f}$ reached by the gas mixture after adiabatic expansion and cooling may therefore be determined as a function of the initial temperature *T* of champagne in the sealed bottles (i.e., before cork popping). Very clearly, and rather counter-intuitively, the higher the initial temperature of champagne before cork popping, the lower the final temperature ${T}_{f}$ reached by the gas mixture after adiabatic cooling, as seen in Fig. 4. By rapidly mixing with adjacent air packages above the bottleneck, holding water vapour with a partial pressure ${P}_{{\rm{vap}}}\approx 0.02\,{\rm{bar}}$ (see the Methods section), ambient air close to the bottleneck cools down. With such a huge drop of temperature of several tens of °C for gas phase expanding out of the bottlenecks, it is no wonder that the temperature of the resulting gas mixture near the bottlenecks falls well beyond the water dew point. It means that locally, and during a very short period of time, ambient air close to the cork popping holds much more water vapour than it can withstand thermodynamically speaking, resulting in the condensation or even freezing of water vapour, as described in the article by Vollmer and Möllman^5^.

### Homogeneous versus heterogeneous nucleation

In the following, focus is made on the gas mixture trapped in the bottlenecks, before, and after adiabatic expansion. Before cork popping, in the sealed bottle, the gas mixture trapped within the bottleneck is indeed mostly composed of gas-phase CO_2_ with only traces of water vapour, as detailed in the Methods section. In the sealed bottle, the strongly temperature-dependent partial pressure of gas-phase CO_2_ is accurately determined through equation (15), whereas the partial pressure of water vapour (being considered as the saturated vapour pressure corresponding to the temperature of storage of the bottle) is given in equation (17). In the range of temperatures between 6 and 20 °C, the partial pressure of gas-phase CO_2_ in the sealed bottles ranges between approximately 4.5 and 7.5 bar (see Fig. 3), whereas the partial pressure of water vapour ranges between about $9\times {10}^{-3}$, and $2.3\times {10}^{-2}\,{\rm{bar}}$. After adiabatic expansion, the pressure within the bottleneck falls to atmospheric pressure (close to 1 bar). CO_2_ being the major component of the gas mixture in the bottleneck, its partial pressure therefore falls close to 1 bar, whatever the bottle temperature, whereas the partial pressure of water vapour falls between about $2\times {10}^{-3}$, and $3\times {10}^{-3}$ bar, depending on the bottle temperature. Moreover, after adiabatic expansion, the huge drop of temperature experienced by the gas mixture found in the bottleneck has some serious effects on the respective saturated vapour pressures of both CO_2_, and water. In the range of very low temperatures reached by the gas mixture after adiabatic expansion (comprised between −76 and −89 °C, depending on the initial temperature of storage before corking), the saturated vapour pressure of CO_2_ ${P}_{{\rm{sat}}}^{{{\rm{CO}}}_{2}}$ is approached through Antoine equation with the appropriate coefficients given in equation (18)^9^. Likewise, in this range of temperatures, the vapour pressure of ice water ${P}_{{\rm{sat}}}^{{{\rm{H}}}_{2}{\rm{O}}}$ was found to obey the relationship displayed in equation (19)^10^.

With the knowledge of the respective gas phase partial pressures of both CO_2_ and water combined with their corresponding saturated vapour pressures, the saturation ratios of both CO_2_ and water (i.e., ${S}_{{{\rm{H}}}_{2}{\rm{O}}}={P}_{{\rm{vap}}}^{{{\rm{H}}}_{2}{\rm{O}}}/{P}_{{\rm{sat}}}^{{{\rm{H}}}_{2}{\rm{O}}}$, and ${S}_{{{\rm{CO}}}_{2}}={P}_{{\rm{vap}}}^{{{\rm{CO}}}_{2}}/{P}_{{\rm{sat}}}^{{{\rm{CO}}}_{2}}$), can be determined in the bottleneck after adiabatic expansion. Depending on the temperature of bottle storage, the saturation ratios of both water and CO_2_ are displayed in Table 1, together with the final temperature reached by the gas mixture in the bottlenecks after adiabatic expansion. Whatever the storage temperature of bottles, the saturation ratio of water vapour reached after adiabatic expansion is huge (${S}_{{{\rm{H}}}_{2}{\rm{O}}} >  > 1$). Phase change from water vapour to ice water is therefore thermodynamically favourable. The situation is different for gas-phase CO_2_. For bottles stored at 6 °C, after adiabatic expansion, the saturation ratio of gas-phase CO_2_ remains lower than unity. CO_2_ is therefore simply unable to undergo a phase change, from gas to dry ice. Nevertheless, for bottles stored at 12 and 20 °C, the saturation ratio of gas-phase CO_2_ goes beyond 1, thus making the bottlenecks a favourable place to freeze gas-phase CO_2_. Actually, as the gas mixture is locally supersaturated with water vapour or gas-phase CO_2_ after adiabatic expansion, the Gibbs free energy term regarding the transfer of molecules from the vapour phase (whether water vapour or gaseous CO_2_) to the solid phase (whether ice water or dry ice) in the form of a cluster of radius *r* is negative. Following the classical nucleation theory (CNT), the nucleation energy barrier ΔG* to overcome, and the corresponding critical radius r* needed for a cluster to spontaneously grow through condensation of water vapour or CO_2_ both express as follows^11, 12^:

2 $$\{\begin{array}{c}{\rm{\Delta }}{G}^{\ast }=\frac{16\pi {\sigma }^{3}{\nu }_{S}^{2}}{3{({k}_{B}T\mathrm{ln}S)}^{2}}\\ {r}^{\ast }=\frac{2\sigma {\nu }_{S}}{{k}_{B}T\,\mathrm{ln}\,S}\end{array}$$

with $\sigma$ being the corresponding surface energy of ice water or dry ice CO_2_, ${\nu }_{S}$ being the corresponding volume of a single molecule in the solid phase, and ${k}_{B}$ being the Boltzmann constant.

**Table 1:** Pertinent parameters of the CO_2_/H_2_O gas mixture found in the bottlenecks, before (in the corked bottles), and after adiabatic expansion.

Storage temperature of bottles (in K)	279	285	293
Pressure of gas-phase CO_2_ in the sealed bottle, ${P}_{{\rm{CB}}}^{{{\rm{CO}}}_{2}}$ (in bar)	4.7	5.8	7.5
Pressure of water vapor in the sealed bottle, ${P}_{{\rm{CB}}}^{{{\rm{H}}}_{2}{\rm{O}}}$ (in bar)	0.0093	0.0140	0.0233
Temperature reached by the gas mixture in the bottleneck after adiabatic expansion, ${T}_{f}$ (in K)	195.7	191.3	183.2
Pressure of gas-phase CO_2_ in the bottleneck after adiabatic expansion, ${P}_{{\rm{vap}}}^{{{\rm{CO}}}_{2}}$ (in bar)	1	1	1
Pressure of water vapor in the bottleneck after adiabatic expansion, ${P}_{{\rm{vap}}}^{{{\rm{H}}}_{2}{\rm{O}}}$ (in bar)	0.0020	0.0024	0.0031
Saturated vapor pressure of gas-phase CO_2_ after adiabatic expansion, ${P}_{{\rm{sat}}}^{{{\rm{CO}}}_{2}}$ (in bar)	1.09	0.70	0.39
Saturated vapor pressure of ice water after adiabatic expansion, ${P}_{{\rm{sat}}}^{{{\rm{H}}}_{2}{\rm{O}}}$ (in Pa)	0.084	0.034	0.011
Saturation ratio of gas-phase CO_2_ after adiabatic expansion, ${S}_{{{\rm{CO}}}_{2}}$	0.92	1.44	2.53
Saturation ratio of water vapor after adiabatic expansion, ${S}_{{{\rm{H}}}_{2}{\rm{O}}}$	2 376	7 023	27 400

According to the CNT, the steady state nucleation rate for homogeneous nucleation ${J}_{\hom }$, defined as the number of clusters that grow past the critical radius ${r}^{\ast }$ per unit volume and per unit time, can be written as^13^:

3 $${J}_{\hom }={N}_{G}\frac{{\rho }_{V}}{{\rho }_{S}}{(\frac{2\sigma }{\pi m})}^{1/2}\exp (-\frac{{\rm{\Delta }}{G}^{\ast }}{{k}_{B}T})$$

with the exponential pre-factor being typically determined from gas-kinetic considerations, *m* being the mass of a single molecule, ${\rho }_{V}$ being the density of the corresponding specie in the gas mixture (water vapour or gas-phase CO_2_), ${\rho }_{S}$ being the density of the solid phase (ice water or dry ice) in the clusters, and ${N}_{G}$ being the molecular concentration of the corresponding specie in the gas mixture (i.e., ${P}_{{\rm{vap}}}^{{{\rm{H}}}_{2}{{\rm{O}}/\text{CO}}_{2}}/{k}_{B}T$, in m^−3^).

As far as homogeneous nucleation is concerned in the bottlenecks after adiabatic expansion, critical radii, nucleation energy barriers, molecular concentrations, and nucleation rates of both water and CO_2_ are presented in Table 2, depending on the initial temperature of bottle storage. Whatever the bottle storage temperature, homogeneous nucleation of ice water clusters is very likely to occur in the bottleneck after adiabatic expansion, given their huge nucleation rates ranging from $\approx {10}^{18}$ cm^−3^ s^−1^ (for bottles stored at 6 °C) to $\approx {10}^{20}$ cm^−3^ s^−1^ (for bottles stored at 20 °C). Inversely, and despite the fact that bottles stored at 12 and 20 °C show saturation ratios significantly higher than 1 for gas-phase CO_2_ after adiabatic expansion, freezing of CO_2_ through homogeneous nucleation remains undoubtedly thermodynamically forbidden, because ${J}_{{\rm{\hom }}}^{{{\rm{CO}}}_{2}}\approx 0$ in both cases. It is indeed well-known that significant amount of homogeneous nucleation requires much higher saturation ratios than those experienced for gas-phase CO_2_ after adiabatic expansion^11, 12^. Compared to homogeneous nucleation, heterogeneous nucleation requires relatively low saturation ratios, but foreign particles or aerosols are needed in the system to initiate the process of phase change by condensing molecules on the pre-existing nuclei. It is nevertheless very unlikely that floating particles, which could promote the freezing of gas-phase CO_2_ through heterogeneous nucleation after adiabatic expansion, pre-exist in the sealed champagne bottlenecks. If eventually present in the bottleneck immediately after corking the bottle, such particles would have been progressively immersed or wetted on the glass wall during the period of aging, before cork popping. However, even in the absence of foreign particles or aerosols pre-existing in a supersaturated condensable environment, heterogeneous nucleation remains possible. Heterogeneous condensation caused by the presence of multiple gaseous species was already described in the literature, particularly in operational rocket plume exhausts that typically consist of mixtures of simple gaseous species^13, 14^. Initial nuclei can be created out of the more easily condensable trace species through homogeneous nucleation, followed by heterogeneous condensation of the less condensable species. In rocket plume exhausts that typically consist of mixtures of simple gaseous species such as N_2_, O_2_, Ar, and CO_2_, condensation can occur when plume temperatures decrease during the expansion process^15–18^. Condensation phenomena with the formation of particles within the plumes can even harm sensitive surfaces of a spacecraft^19, 20^. In the article by Li *et al*.^13^, simulations of homogeneous and heterogeneous condensations were performed to study freely expanding mixtures of CO_2_ and N_2_ condensation plumes. A pure N_2_ expanding flow was found to not produce any clusters, whereas in a mixture consisting of 5% CO_2_ and 95% N_2_, under the same expansion conditions, heterogeneous condensation of N_2_ molecules on homogeneously condensed CO_2_ nuclei was reported.

**Table 2:** Based on the classical nucleation theory (CNT), critical radii, nucleation energy barriers, and corresponding homogeneous nucleation rates of both water and CO_2_ after adiabatic expansion. To evaluate the critical radii, homogeneous nucleation energy barriers, and nucleation rates of both ice water and dry ice CO_2_ clusters, their respective surface energy, and density were used (i.e., ${\sigma }_{{{\rm{H}}}_{2}{\rm{O}}}\approx 0.106\,{\rm{J}}\,{{\rm{m}}}^{-2}$, ${\sigma }_{{{\rm{C}}{\rm{O}}}_{2}}\approx 0.08\,{\rm{J}}\,{{\rm{m}}}^{-2}$, ${\rho }_{{{\rm{H}}}_{2}{\rm{O}}}\approx 920\,{\rm{kg}}\,{{\rm{m}}}^{-3}$, and ${\rho }_{{{\rm{CO}}}_{2}}\approx 1600\,{\rm{kg}}\,{{\rm{m}}}^{-3}$ ^29^).

Storage temperature of bottles (in K)	279	285	293
Saturation ratio of gas-phase CO_2_ after adiabatic expansion, ${S}_{{{\rm{CO}}}_{2}}$	0.92	1.44	2.53
Saturation ratio of water vapor after adiabatic expansion, ${S}_{{{\rm{H}}}_{2}{\rm{O}}}$	2 376	7 023	27 400
Critical radius for water clusters after adiabatic expansion, ${r}_{{{\rm{H}}}_{2}{\rm{O}}}^{\ast }$ (in m)	$2.9\times {10}^{-10}$	$2.6\times {10}^{-10}$	$2.3\times {10}^{-10}$
Critical radius for CO_2_ clusters after adiabatic expansion, ${r}_{{{\rm{CO}}}_{2}}^{\ast }$ (in m)	/	$7.7\times {10}^{-9}$	$3.1\times {10}^{-9}$
Nucleation energy barrier for water clusters after adiabatic expansion, ${\rm{\Delta }}{G}_{{{\rm{H}}}_{2}{\rm{O}}}^{\ast }$ (in J)	$3.4\times {10}^{-20}$	$2.8\times {10}^{-20}$	$2.2\times {10}^{-20}$
Nucleation energy barrier for CO_2_ clusters after adiabatic expansion, ${\rm{\Delta }}{G}_{{{\rm{CO}}}_{2}}^{\ast }$ (in J)	/	$2.0\times {10}^{-17}$	$3.3\times {10}^{-18}$
Molecular concentration of water vapor in the bottleneck after adiabatic expansion, ${N}_{{\rm{G}}}^{{{\rm{H}}}_{2}{\rm{O}}}$ (in molecules cm^−3^)	$7.4\times {10}^{16}$	$9.2\times {10}^{16}$	$1.2\times {10}^{17}$
Molecular concentration of gas-phase CO_2_ after adiabatic expansion, ${N}_{{\rm{G}}}^{{{\rm{CO}}}_{2}}$ (in molecules cm^−3^)	$3.7\times {10}^{19}$	$3.8\times {10}^{19}$	$3.9\times {10}^{19}$
Homogeneous nucleation rate for water clusters after adiabatic expansion, ${J}_{{\rm{\hom }}}^{{{\rm{H}}}_{2}{\rm{O}}}$ (in cm^−3^ s^−1^)	$8\times {10}^{17}$	${10}^{19}$	${10}^{20}$
Homogeneous nucleation rate for CO_2_ clusters after adiabatic expansion, ${J}_{{\rm{\hom }}}^{{{\rm{CO}}}_{2}}$ (in cm^−3^ s^−1^)	/	$\approx 0$	$\approx 0$

At a smaller scale indeed, we believe that champagne bottlenecks could be viewed as small rocket nozzles. By drawing a parallel between the gas mixture freely expanding during the champagne cork popping process, and condensation phenomena observed in freely expanding condensation plumes, we therefore propose the following scenario. After adiabatic expansion of the gas mixture following the cork popping process of champagne bottles, clusters of ice water appear in the bottlenecks through homogeneous nucleation due to the very high saturation ratio experienced by water vapour, whatever the storage temperature of bottles. For bottles stored at 6 °C, the saturation ratio of gas-phase CO_2_ nevertheless remains lower than 1, thus simply forbidding the freezing of CO_2_ (whether through homo- or heterogeneous nucleation). The amount of water vapour being very low in the bottlenecks, the bottleneck remains optically transparent. For bottles stored at 12 and 20 °C, the saturation ratio of gas-phase CO_2_ is significantly higher than 1, thus enabling the freezing of gas-phase CO_2_ (through heterogeneous nucleation only) on ice water cluster nuclei. Blue haze is therefore attributed to the freezing of gas-phase CO_2_ on ice water nuclei much smaller than the wavelength of light. Moreover, heterogeneous freezing of gas-phase CO_2_ on ice water nuclei starts earlier, and with a much stronger effect for the bottles stored at 20 °C showing the highest saturation ratio (as clearly observed in Fig. 1b,c).

### Rayleigh scattering

Blue haze is typical of Rayleigh scattering, which describes the elastic scattering of light by spherical particles much smaller than the wavelength of light. At the wavelength $\lambda$, for spherical particles with radii *a*, and with a refractive index *n*, the Rayleigh scattering cross-section ${\sigma }_{R}$ is given by the following relationship^7^:

4 $${\sigma }_{R}=\frac{128{\pi }^{5}{a}^{6}}{3{\lambda }^{4}}{(\frac{{n}^{2}-1}{{n}^{2}+2})}^{2}$$

**Figure 5:**
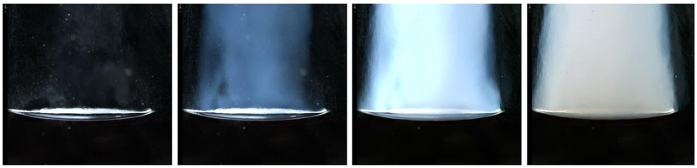
After adiabatic expansion, in the bottleneck of bottles stored at 20 °C, the progressive growth in size of dry ice CO_2_ clusters can also be evidenced by observing the change in colour experienced by the condensation cloud, which progressively changes from deep blue to grey-white. The time interval between each frame is 83 µs.

At the wavelength $\lambda \approx 0.4$ μm (which corresponds to the blue region of the visible light spectrum), dry ice CO_2_ has a refractive index $n\approx 1.35$ ^21^. Therefore, considering *a* in the latter equation as being the critical radius for dry ice CO_2_ clusters after adiabatic expansion for bottles stored at 20 °C (see Table 2) yields to a cross-section ${\sigma }_{R}\approx 1.7\times {10}^{-23}{{\rm{m}}}^{2}$. This result has to be compared to the Rayleigh scattering cross-section of ambient air surrounding the bottlenecks during our observations. Indeed, and similarly to the Earth atmosphere, ambient air in the laboratory does also scatter light. By keeping the wavelength $\lambda \approx 0.4$ μm, and by using an equation provided by the literature for atmospheric scattering^7^, the cross-section for ambient air was found to be ${\sigma }_{{\rm{air}}}\approx 1.7\times {10}^{-30}\,{{\rm{m}}}^{2}$ (i.e., seven orders of magnitude lower than the scattering cross-section of dry ice CO_2_ clusters). Therefore, despite the fact that the number of CO_2_ clusters per unit volume is still unknown, this huge ratio of order of 10^7^ between ${\sigma }_{R}$ and ${\sigma }_{{\rm{air}}}$ tells us that even a modest number of dry ice CO_2_ condensation nuclei would be enough to produce a much stronger scattering in the blue than ambient air (as observed during the cork popping of bottles stored at 20 °C, where gas-phase CO_2_ is strongly suspected to freeze in the bottlenecks). We therefore conclude that this characteristic blue haze is the signature of a partial and transient freezing of gas-phase CO_2_ initially present in the bottleneck before cork popping. After adiabatic expansion, the progressive growth in size of dry ice CO_2_ clusters can even be evidenced by observing the change in colour of the condensation cloud found in the bottleneck of bottles stored at 20 °C, as shown in the time sequence displayed in Fig. 5. It is worth noting that the cloud colour progressively changes from deep blue to white-grey, which pleads in favour of a transition between Rayleigh scattering by nuclei much smaller than the wavelength of ambient light, and Mie scattering as the size of nuclei becomes comparable and larger than the wavelength of light.

### Inhibition of water vapour condensation above the bottlenecks

**Figure 6:**
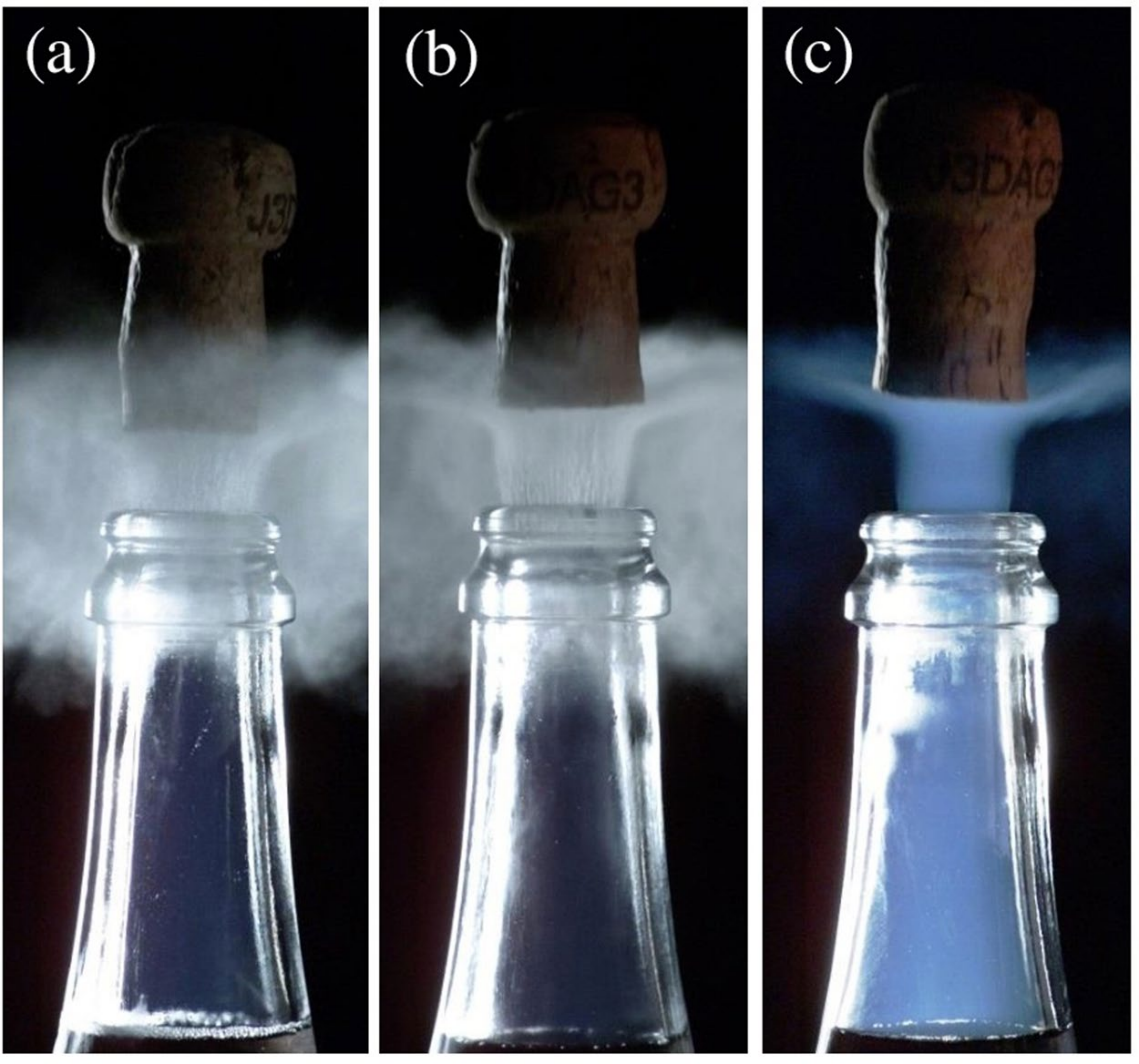
Three snapshots, taken 1.2 ms after the cork popping process, showing the condensation of water vapour above the bottlenecks of bottles stored at 6 °C (**a**), 12 °C (**b**), and the deep blue CO_2_ freezing plume gushing from the bottleneck of the bottle stored at 20 °C (**c**), respectively. In frame (**c**), the grey-white cloud of condensation droplets found in air packages adjacent to the gas volume gushing out of the bottleneck disappeared.

As already mentioned earlier, the grey-white plume above the bottlenecks (clearly observed for bottles stored at 6 and 12 °C) is the signature of the condensation of water vapour naturally present in ambient air^2, 5, 6^. The other striking feature revealed by our experiments is the complete disappearance of this grey-white plume above the bottleneck of champagne stored at 20 °C, while the blue haze starts early within the bottleneck, as exemplified in Fig. 6. It is worth noting that the energy required to condense water vapour is brought by the change of internal energy ${\rm{\Delta }}U$ of the gas mixture initially found in the bottlenecks. The first law of thermodynamics states that ${\rm{\Delta }}U=Q+W$, where $Q$ denotes the exchange of heat during the process, and $W$ relates to the work of expansion of the gas mixture gushing out of the bottleneck. For adiabatic processes, *Q* = 0, so that the gas mixture experiences a drop of its internal energy determined by the following relationship:

5 $${\rm{\Delta }}U={\int }_{{V}_{G}}^{{V}_{f}}PdV$$

with *P* being the pressure of the gas mixture freely expanding during adiabatic expansion, ${V}_{G}$ being the volume of the gas mixture in the sealed bottle before cork popping, and ${V}_{f}$ being the volume of the gas mixture after adiabatic expansion.

Adiabatic expansion keeping the product $P{V}^{\gamma }$ as constant, the latter equation therefore transforms as follows:

6 $${\rm{\Delta }}U={P}_{CB}{V}_{G}^{\gamma }{\int }_{{V}_{G}}^{{V}_{f}}\frac{dV}{{V}^{\gamma }}$$

with ${P}_{CB}$ being the strongly temperature dependent pressure of gas-phase CO_2_ in the corked bottle.

Integrating equation (6) between the initial stage in the corked bottle, and the final stage after adiabatic expansion, and developing, leads to the following relationship, function of both the initial pressure, and the volume of gas phase in the corked bottle:

7 $${\rm{\Delta }}U=\frac{{P}_{CB}{V}_{G}}{(\gamma -1)}[1-{(\frac{{P}_{CB}}{{P}_{0}})}^{\frac{1-\gamma }{\gamma }}]$$

By application of the latter equation to the bottles stored at 6 °C, and with a pressure in the sealed bottles in the order of 4.5 bar, the decrease in internal energy of the gas mixture after adiabatic expansion is in the order of ${\rm{\Delta }}U\approx 11\,{\rm{J}}$. A fraction of this energy is used to cool adjacent air packages from about 20 °C to the dew point temperature (around 14.5 °C for ambient air with ${\rm{RH}}\approx 70 \%$). Then another fraction of this energy is required to force phase change by condensing a portion of water vapour in the form of liquid droplets, and thus forming the very characteristic grey-white cloud of fog above the bottleneck. By application of the latter equation to the bottles stored at 20 °C, and with a pressure in the sealed bottles in the order of 7.5 bar, the decrease in internal energy of the gas mixture after adiabatic expansion becomes in the order of ${\rm{\Delta }}U\approx 24\,{\rm{J}}$. Actually, because the saturation ratio of gas-phase CO_2_ becomes in the order of 2.5 after adiabatic expansion, a fraction of gas-phase CO_2_ is believed to be able to experience gas phase and transform into dry ice CO_2_ (through heterogeneous nucleation on ice water cluster nuclei, as proposed earlier). But since the latent heat of sublimation of dry ice CO_2_ is high (with ${\rm{\Delta }}{H}_{{\rm{sub}}}^{{{\rm{CO}}}_{2}}\approx 26\,{\rm{kJ}}\,{{\rm{mol}}}^{-1}$ ^22^), phase change of even a small portion of CO_2_ from gas phase to dry ice will pump the loss of internal energy ${\rm{\Delta }}U\approx 24\,{\rm{J}}$ released through adiabatic expansion. For bottles stored at 20 °C, about $8\times {10}^{-3}\,{\rm{mol}}$ of gas-phase CO_2_ experience adiabatic expansion. It is worth noting that the deposition of only about $9\times {10}^{-4}\,{\rm{mol}}$ of gas-phase CO_2_ requires an energy of $Q\approx 24\,{\rm{J}}$. Therefore, the phase change from gas phase to dry ice of only a small volume fraction of all the available CO_2_ (in the order of 0.1) is able to pump all the available energy released through adiabatic expansion. Finally, the freezing of only about 10% of the gas-phase CO_2_ found in the bottleneck can forbid the condensation of water vapour found in air packages adjacent to the gas volume gushing out of the bottleneck, as observed during the cork popping process of bottles stored at 20 °C.

## Conclusion

Initially under pressure in the bottleneck, a gas mixture mainly composed of gas-phase CO_2_ with traces of water vapour freely expands during cork popping. The commonly accepted idea behind champagne cork popping was that the gas mixture gushing from the bottleneck experiences adiabatic expansion, and therefore cools adjacent air packages, thus causing condensation of water vapour found in ambient air in the form of a characteristic grey-white cloud of fog. Nevertheless, based on this set of experiments, the situation has appeared really more complex and subtle. We pointed out the key role of bottle temperature. Most interestingly, for the bottles stored at 20 °C, the characteristic grey-white cloud of fog classically observed above the bottlenecks of bottles stored at lower temperatures completely disappeared. It was replaced by a more evanescent plume, surprisingly blue, starting from inside the bottleneck. Depending on the strongly bottle temperature dependent saturation ratio experienced by gas-phase CO_2_ after adiabatic expansion, it was emphasized that blue haze is the signature of a partial and transient heterogeneous freezing of gas-phase CO_2_ on ice water clusters homogeneously nucleated in the bottlenecks.

## Materials and Methods

### Batch of champagne bottles

A single batch of champagne rosé (from Champagne Vranken Pommery, Marne, France), elaborated with a blend of chardonnay and pinot noir base wines (vintage 2008), was used for this set of experiments. In March 2010, the blended wine was put in standard 75 cL clear transparent bottles, along with yeasts and sugar to promote its second fermentation (or *prise de mousse*). A classical amount of 24 g L^−1^ of sugar was added in the blend to promote the *prise de mousse* ^3^. Bottles were then hermetically sealed with a crown cap. The key metabolic process behind the production of gaseous CO_2_ in the sealed bottles is alcoholic fermentation (i.e., the conversion of sugars into ethanol and gaseous carbon dioxide by yeast), displayed hereafter.

8 $${{\rm{C}}}_{6}{{\rm{H}}}_{12}{{\rm{O}}}_{6}\to 2\,{{\rm{CH}}}_{3}{{\rm{CH}}}_{2}{\rm{OH}}+2\,{{\rm{CO}}}_{2}$$

Following equation (8) with 24 g L^−1^ of sugar added in the blend, leads to the production of 9 g of CO_2_ (i.e., 0.2 mole) in every bottle of this batch during the *prise de mousse*.

### Batch of cork stoppers

After a long period of aging of 42 months following the *prise de mousse*, bottles were then disgorged (in order to remove the sediment of dead yeast cells), and corked with traditional natural cork stopper from a same batch of corks (provided by Amorim & Irmãos, Portugal). Each cork stopper is composed of two well-distinct different parts: (i) a upper part composed of agglomerated cork granules, and (ii) a lower part, into contact with the wine, made of two massive cork disks stuck together. The mass of each cork is about 10 ± 0.1 g. It is also worth noting that, into every bottle of this batch, the volume of the headspace under the cork is precisely equal to 25 mL. To examine the role of temperature on champagne cork popping, three storage temperatures were chosen in a reasonable range of temperatures likely to be encountered by bottles during champagne making or even by standard consumers (namely, 6, 12, and 20 °C, respectively). 72 hours before each set of experiments, bottles were stored at the desired temperature.

### Temperature dependence of gas phase pressure in the corked bottles

The capacity of CO_2_ to get dissolved in champagne is ruled by Henry’s law equilibrium, which states that the concentration *c* of dissolved CO_2_ in the liquid phase is proportional to the partial pressure of gas-phase CO_2_ denoted *P*:

9 $$c={k}_{H}P$$

with ${k}_{H}$ being the strongly temperature-dependent Henry’s law constant of gas-phase CO_2_ in the liquid phase (i.e., its solubility)^23, 24^.

Thermodynamically speaking, the temperature-dependence of Henry’s law constant can be conveniently expressed with a van’t Hoff like equation as follows:

10 $${k}_{H}(T)={k}_{298{\rm{K}}}\exp [-\frac{{\rm{\Delta }}{H}_{{\rm{diss}}}}{R}(\frac{1}{T}-\frac{1}{298})]$$

with ${\rm{\Delta }}{H}_{{\rm{diss}}}$ being the dissolution enthalpy of CO_2_ molecules in the liquid phase, and *R* being the ideal gas constant ($8.31\,{\rm{J}}\,{{\rm{K}}}^{-1}{{\rm{m}}{\rm{o}}{\rm{l}}}^{-1}$). The best fit of ${k}_{H}(T)$ with a previous set of experimental data was found with ${k}_{298{\rm{K}}}\approx 1.21\,{\rm{g}}\,{{\rm{L}}}^{-1}{{\rm{b}}{\rm{a}}{\rm{r}}}^{-1}$, and ${\rm{\Delta }}{H}_{{\rm{diss}}}\approx 24.8\,{\rm{kJ}}\,{{\rm{mol}}}^{-1}$ ^25^.

In the sealed bottle, the volume ${V}_{G}$ of gas phase in the headspace cohabits with a volume ${V}_{L}$ of champagne (i.e., the liquid phase). For the sake of simplicity, we suppose that both volumes remain constant during the *prise de mousse* (i.e., we neglect the minute changes of the liquid volume due to the progressive dissolution of CO_2_). In the pressure range of interest (a few bars), we may safely suppose that the gas phase is ruled by the ideal gas law. Thus,

11 $$P{V}_{G}={n}_{G}RT$$

with *T* being the champagne temperature (in K), and ${n}_{G}$ being the mole number of gas-phase CO_2_ in the headspace.

Moreover, in the bottle hermetically sealed, the total number of moles of carbon dioxide ${n}_{T}$ is a conserved quantity that decomposes into ${n}_{G}$ moles in the gaseous phase and ${n}_{L}$ moles in the liquid phase. Therefore,

12 $${n}_{T}={n}_{G}+{n}_{L}$$

By combining equations (9), (11) and (12), the following relationship was determined for the pressure of gas-phase CO_2_ in the sealed bottle after the *prise de mousse* was achieved:

13 $${P}_{PDM}\approx \frac{{n}_{T}RT}{{V}_{G}+{k}_{H}RT{V}_{L}}$$

with ${P}_{PDM}$ being expressed in Pa, and ${k}_{H}$ being conveniently expressed in ${\rm{mol}}\,{{\rm{m}}}^{-3}\,{{\rm{Pa}}}^{-1}$.

After the *prise de mousse*, bottles aged in a cool cellar for 42 months. Bottles were then disgorged, which consists in opening the bottles in order to remove the dead yeast cells, and re-corking them with the traditional cork stoppers described above. During the disgorging process, a bit of CO_2_ is inevitably lost at this step as gas-phase CO_2_ escapes from the bottleneck under the action of pressure when opening the bottle. The partial pressure of gas-phase CO_2_ therefore falls. The thermodynamic equilibrium of CO_2_ is broken, but the bottle is then quickly re-corked with a traditional cork stopper. Dissolved and gas-phase CO_2_ therefore quickly recover Henry’s equilibrium in the corked bottle. The newly defined total number of CO_2_ moles in the re-corked bottle, namely ${n}_{T}^{CB}$, finally becomes equivalent to:

14 $${n}_{T}^{CB}={n}_{T}-{n}_{G}\approx {n}_{T}-\frac{{P}_{PDM}{V}_{G}}{RT}$$

The newly recovered equilibrium pressure of gas-phase CO_2_ is therefore accessed by replacing ${n}_{T}$ in equation (13) with the newly defined total number of CO_2_ moles in the corked bottle ${n}_{T}^{CB}$, defined in equation (14). Finally, and after developing, the newly recovered equilibrium pressure of gas-phase CO_2_ can be expressed as:

15 $${P}_{CB}\approx \frac{{n}_{T}{k}_{H}{(RT)}^{2}{V}_{L}}{{({V}_{G}+{k}_{H}RT{V}_{L})}^{2}}$$

For standard champagne bottles with ${V}_{L}=75\,{\rm{cL}}$, a gaseous volume in the headspace of ${V}_{G}=25\,{\rm{mL}}$, and a total number of CO_2_ moles trapped per bottle of ${n}_{T}\approx 0.2\,{\rm{mole}}$ (after a *prise de mousse* promoted with 24 g L^−1^ of sugar), the temperature dependence of both gas-phase CO_2_ pressures ${P}_{PDM}$, and ${P}_{CB}$, as given in equations (13) and (15), respectively, is displayed in Fig. 3. The disgorging step has therefore a slight but significant impact on the final pressure found within the corked bottle (especially near ambient temperatures), and has therefore to be taken into account in every problematic dealing with pressure dependence phenomena. It was indeed wrongly neglected in previous studies dedicated to thermodynamic equilibrium of CO_2_ within the sealed bottles^4, 26^.

### High-speed video device

A digital high-speed cinema camera (Phantom Flex - Vision Research, USA) was used for this set of experiments on the visualization of phenomena accompanying champagne cork popping. Classically, the maximum filming rate of the Phantom Flex digital camera increases as its resolution decreases, with for example up to 2.570 frames per second (fps) at a resolution of 1920 × 1080 pixels, 5.350 fps at 1280 × 720, and 10.750 fps at 640 × 480. Generally speaking, powerful illumination is required to get satisfying high frame rate moving time sequences. A 5000 W lamp using tungsten filaments, and powered by direct current (DC), was placed behind the corked bottleneck. It is worth noting that, in addition to a powerful illumination, DC is needed to eliminate the so-called flicker phenomenon. Actually, when powered by 60 Hz alternating current (AC) electricity, the lamp tungsten filament intensity can slightly vary with the same frequency, thus altering time sequences above a 120 fps frame rate. To get synchronized with the split-second timing of cork popping, the shooting of the camera was post-triggered by using a simple microphone recording the “bang” done by the fast traveling cork popping out of the bottleneck. The powerful lamp, which acts as a source of heat, was switched on just before filming the cork popping process, and switched off immediately after recording the sequence.

### Relative humidity in ambient air

Temperature and hygrometry of ambient air were supplied with a digital coupled Thermo-Hygrometer (2212TM, IHM, France). Our cork popping experiments were done in a controlled temperature room (at 20 ± 2 °C), where the relative humidity (RH) slightly varied throughout the day, but was very close to 70%. At a given temperature *T*, RH is defined as follows:

16 $${\rm{RH}}=S\times 100=\frac{{P}_{{\rm{vap}}}}{{P}_{{\rm{sat}}}(T)}\times 100$$

with *S* being the saturation water vapour ratio, ${P}_{{\rm{vap}}}$ being the partial pressure of water vapour in air, and ${P}_{{\rm{sat}}}(T)$ being the strongly temperature-dependent saturated vapour pressure.

In ambient air, ${P}_{{\rm{sat}}}(T)$ is correctly approached through the Clausius-Clapeyron equation defined as follows, provided that water vapour behaves as an ideal gas, and that the specific latent heat of water evaporation ${L}_{{\rm{vap}}}$ remains reasonably constant in this range of temperatures^27^:

17 $${P}_{{\rm{sat}}}(T)={P}_{0}\exp [\frac{M{L}_{{\rm{vap}}}}{R}(\frac{1}{373}-\frac{1}{T})]$$

with ${P}_{0}$ being the ambient pressure (close to 1 bar), *M* being the molar mass of water (0.018 kg mol^−1^), and ${L}_{{\rm{vap}}}$ being the specific latent heat of water evaporation at 20 °C ($\approx 2.47\times {10}^{6}\,{\rm{J}}\,{{\rm{kg}}}^{-1}$)^28^.

Therefore, by combining ${\rm{RH}}\approx 70 \%$ (at $T\approx 20\,^\circ {\rm{C}}$) with equations (16) and (17) enabled to retrieve the partial pressure of water vapor in ambient air as ${P}_{{\rm{vap}}}\approx 0.02\,{\rm{bar}}$ during the day our cork popping experiments were done.

### Temperature dependence of ice water and dry ice CO_2_ saturated vapor pressures

The relationship between the saturated vapor pressure of gas-phase CO_2_ ${P}_{{\rm{sat}}}^{{{\rm{CO}}}_{2}}$, and temperature *T* is provided by Antoine equation as follows^9^:

18 $${\mathrm{log}}_{10}({P}_{{\rm{sat}}}^{{{\rm{CO}}}_{2}})=A-\frac{B}{T+C}$$

with ${P}_{{\rm{sat}}}^{{{\rm{CO}}}_{2}}$ being expressed in bar, *T* being expressed in K, and the coefficients *A*, *B*, and *C* being provided by the NIST database^28^. In the range of temperatures between 154 and 196 K (i.e., between −119 and −77 °C), that covers the range of temperatures reached by the gas mixture gushing out of the bottleneck after adiabatic expansion, Antoine coefficients *A*, *B*, and *C* are 6.81228, 1301.679, and −3.494, respectively^28^.

Likewise, in this range of temperatures, the vapor pressure of ice water ${P}_{{\rm{sat}}}^{{{\rm{H}}}_{2}{\rm{O}}}$ was found to obey the following relationship^10^:

19 $${P}_{{\rm{sat}}}^{{{\rm{H}}}_{2}{\rm{O}}}=\exp (28.868-\frac{6132.9}{T})$$

with ${P}_{{\rm{sat}}}^{{{\rm{H}}}_{2}{\rm{O}}}$ being expressed in Pa, and *T* being expressed in K.
